# Visualization of gene expression in *Pristionchus pacificus* with smFISH and in situ HCR

**DOI:** 10.17912/micropub.biology.001274

**Published:** 2024-07-26

**Authors:** Yasmin H. Ramadan, Oliver Hobert

**Affiliations:** 1 Biological Sciences, Columbia University, New York, United States; 2 Biological Sciences, Columbia University, Howard Hughes Medical Institute, New York, United States

## Abstract

Single molecule fluorescence in situ hybridization (smFISH) and in situ hybridization chain reaction (HCR) have become powerful tools to visualize gene expression in many different animal species. We show here that smFISH and in situ HCR can be put to effective use in the satellite nematode model organism *Pristionchus pacificus .* Examining the expression of a homeobox gene ( *Ppa-unc-30)* , we found that HCR is more sensitive than smFISH. We confirmed the robustness of HCR by visualization of the expression of several genes involved in neurotransmitter synthesis or transport ( *Ppa-unc-25 /GAD, Ppa-unc-17/VAChT, Ppa-eat-4 /VGLUT).* Combined with its relative cost-effectiveness compared to smFISH analysis, in situ HCR constitutes a useful addition to the toolbox for *P. pacificus* research *.*

**Figure 1. smFISH and in situ HCR in P. pacificus:**
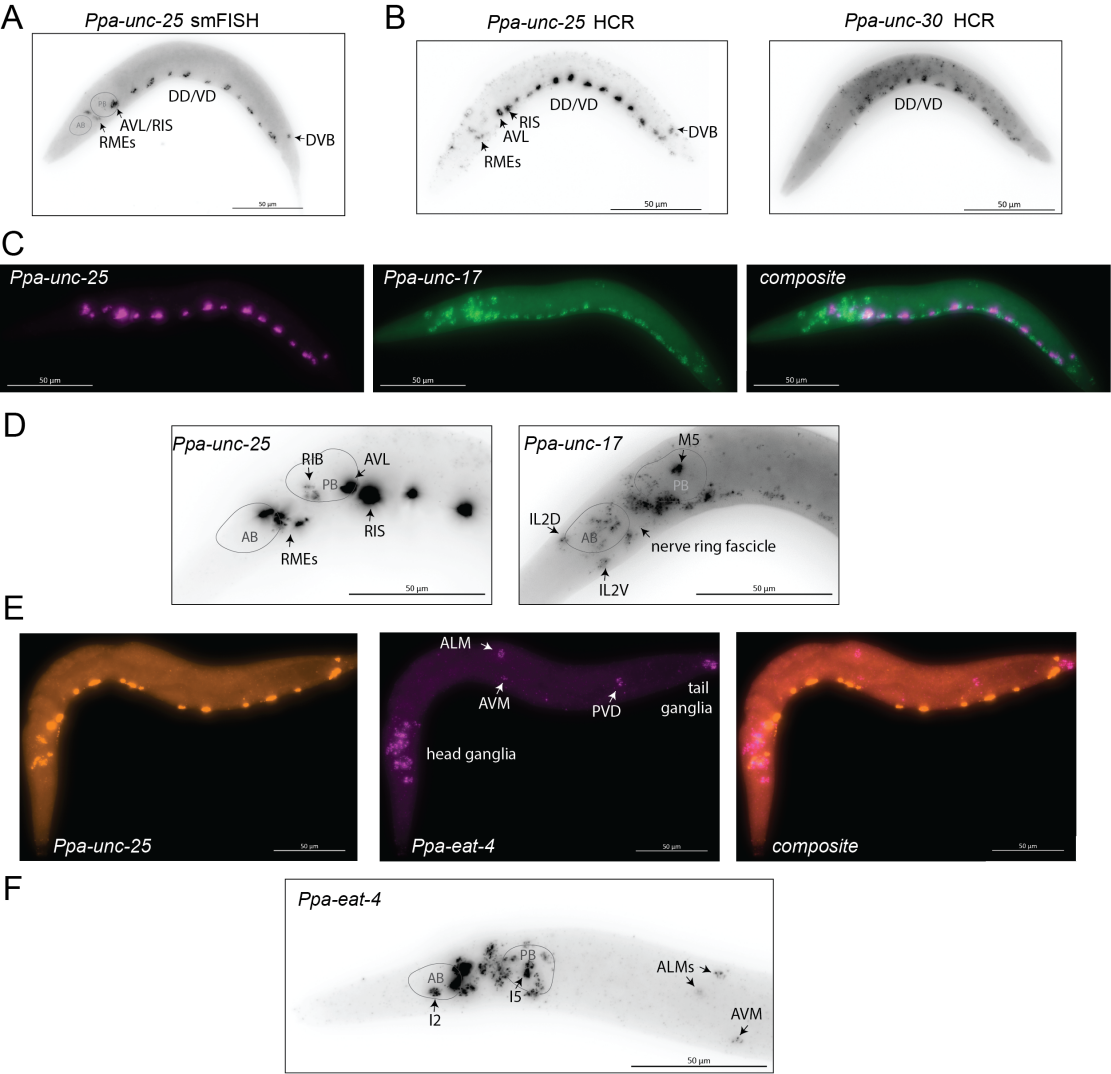
Cellular identities shown in all panels are tentative and are based on the identity of *C. elegans* neurons that express the respective gene in roughly the same position. Further confirmation is required to solidify these assignments. All images shown are maximum z-stack projections. Scale bar = 50µm. A: smFISH showing expression of *Ppa-unc-25* in a J2 larval animal. AB/PB: location of anterior/posterior bulb of pharynx. B: HCR probing of *Ppa-unc-25* (left) and *Ppa-unc-30* (right) gene expression in a J2 larval animal. C,D: HCR probing of *Ppa-unc-17* and *Ppa-unc-25* gene expression in J2 (C) and J3 (D) larval animals. E,F: HCR probing of *Ppa-eat-4* and *Ppa-unc-25* gene expression patterns in J3 larval animals.

## Description

The nematode *Pristionchus pacificus* is a powerful satellite model organism for comparative evolutionary studies (Sommer and McGaughran 2013) . One key component to the success of comparative analysis is the ability to reliably assess gene expression patterns with spatial and temporal specificity in whole animals. While fluorescent reporter transgenes can be generated with ease in *C. elegans ,* they are more difficult to generate in *P. pacificus* , even though substantial strides have been made in improving the efficiency of *P. pacificus* transgenesis (Schlager *et al.* 2009; Namai and Sugimoto 2018; Han *et al.* 2020).

Single molecule fluorescence in situ hybridization (smFISH) represents an alternative means to visualize gene expression (Ji and van Oudenaarden 2012) , but the high costs of probe generation restrict the attractiveness of this technique. The more recent introduction of in situ hybridization chain reaction (HCR) represent a more cost-effective approach for the *in situ* analysis of gene expression (Choi *et al.* 2016). The greater cost-effectiveness stems from the ability to use unconjugated oligonucleotide sequences as probes. Each probe set consists of 25bp probes generated from complementary sequences within a gene's locus and a 18bp initiator sequence unique to an amplifier (B1, B2, B3, etc.). The fluorophores used to visualize probe-target hybridization are conjugated to a hairpin sequence that is not gene-specific and thus may be used for multiple experiments. HCR has been applied in many different organisms, including *C. elegans* (Choi *et al.* 2016) and we set out here to test the usage of HCR, as well as of smFISH, in *P. pacificus.*

We generated smFISH probes for two *P. pacificus* genes, *Ppa-unc-25* (GAD) *,* encoding glutamic acid decarboxylase, the rate limiting enzyme for synthesis of the neurotransmitter GABA (Eastman *et al.* 1999) and *Ppa-unc-30,* a Pitx-type homeobox transcription factor whose *C. elegans* ortholog controls *Ppa-unc-25* expression in GABAergic ventral nerve cord neurons (Jin *et al.* 1994). We were able to readily detect *Ppa-unc-25* transcripts in a pattern very similar to what is observed in *C. elegans ,* namely in the ventral nerve cord and in several head neurons, including what appear to be the RME, RIS, RIB and AVL neurons ( **Figure 1A** ). However, we failed to detect any signals with smFISH probes for the *Ppa-unc-30* locus. Moreover, *Ppa-unc-25* smFISH probing was unstable, requiring worms to be immediately imaged following the protocol to avoid risk of losing signal altogether.

We generated HCR probes for the same two genes ( *Ppa-unc-25 , Ppa-unc-30)* and followed a protocol that has been put to use in *C. elegans* (see Methods section)(Choi *et al.* 2016). We observed more robust signals with the *Ppa-unc-25* HCR probes, and we now also observed signals with the *Ppa-unc-30* HCR probes in the ventral nerve cord, as well as several head neurons ( **Figure 1B** ).

We further probed the generality of HCR usage by generating probes to two other genes, the single *Ppa-eat-4 /VGLUT* locus to reveal glutamatergic neurons and the single *Ppa-unc-17/VAChT* locus to reveal cholinergic neurons. We generated these two probes with different amplifier sequences to allow us to co-stain the expression of each gene relative to the expression of *Ppa-unc-25 /GAD* . As expected from studies in *C. elegans* (Serrano-Saiz *et al.* 2013; Pereira *et al.* 2015; Gendrel *et al.* 2016), we observed non-overlapping expression of *Ppa-unc-25 /GAD* compared to *Ppa-eat-4 /VGLUT* and compared to *Ppa-unc-17/VAChT* ( **Figure 1 C -F** ) *.* The patterns of *Ppa-eat-4 /VGLUT* and *Ppa-unc-17/VAChT* expression in *P. pacificus* appear roughly comparable to those observed for their respective *C. elegans* homologs (Serrano-Saiz *et al.* 2013; Pereira *et al.* 2015) but future studies will be required to pinpoint the exact identity of the *Ppa-eat-4 /VGLUT* and *Ppa-unc-17/VAChT* expressing neurons. Since mRNA signals are unevenly distributed throughout the soma of a given cell, it is difficult to separate signals from neighboring cells within densely packed ganglia. We envision that the problem of cell identification will be solved by multicolor co-staining with co-expressed genes.

We note that smFISH and HCR appear to offer several advantages over antibody staining. Due to antibody penetration issues, immunostaining protocols usually only stain a fraction of the animals in the sample, often with a strong bias for larval stage animals. On the other hand, we note that smFISH and HCR protocols produce signals in nearly 100% of larval and adult stage animals. Additionally, many antibody staining protocols require harsh steps to fix and permeabilize the *C. elegans* cuticle (Duerr 2006; Shakes *et al.* 2012; Gendrel *et al.* 2016), which tend to distort and disintegrate the animal, particularly in adults, and therefore complicate the accurate visualization of gene expression patterns.

In conclusion, the cost-effectiveness, robustness, and sensitivity of the HCR protocol will empower *P. pacificus* researchers to analyze gene expression profiles in this organism with relative ease.

## Methods

For smFISH staining, we followed the smFISH protocol for *C. elegans* described in WormBook (Ji and van Oudenaarden 2012) .

For HCR, we followed the protocol described in Choi et al., 2016, with a number of slight changes. Below, we describe the Choi et al. protocol, including our adaptations:

Probe Design:

1. ‘HCR probe maker', a probe design software by the Ozplut lab, was downloaded from the following (Kuehn et al., 2022):

a. For Mac: https://github.com/rwnull/HCRProbeMakerCL/tree/HCRProbeMakerCLforMac

b. For PC: https://github.com/rwnull/insitu_probe_generator

2. Probes were designed according to the guidelines described in Choi et al, 2016: the first probe in a pair began with an initiator sequence (B1, B2, or B3, see below), followed by the 2bp spacer ‘tt', then a 25bp complimentary sequence. The second probe in a pair began with the adjacent complementary sequence, followed by the same spacer and initiator sequence. The following adaptations were made for the aforementioned experiments:

a. Probe sets were designed such that the maximum number of probes were generated per gene.

b. Initiator sequences were chosen such that *Ppa-unc-25* could be uniquely labeled in a different color with every other gene investigated.

**Table:** 

***Gene***	***Number of probe pairs***	***Initiator sequence used (conjugated fluorophore)***
*Ppa-* ***unc-25***	26	B1 (AF 647), B2 (AF 546)
*Ppa-* ***unc-30***	14	B2 (AF 546)
*Ppa-* ***unc-17***	28	B3 (AF 488)
*Ppa-* ***eat-4***	32	B1 (AF 647)

3. Hairpin sequences for each initiator used (B1, B2, and B3) were ordered from molecularinstruments.com, conjugated with AF 647, AF 546, and AF 488, respectively.

4. Probes were ordered from IDT as DNA Oligo Pools (50µM tubes). Probes were reconstituted in 50 µL of NF-free H _2_ O for a 1µM stock and stored at -20˚C for long-term storage.

Fixation :

NOTE: Reagents, equipment & bench space were kept diligently RNAse free throughout.

1. Worms were washed off plates with 1 mL of M9 buffer and transferred to a 15 mL conical tube. To ensure high yield (worms will be lost throughout successive washes/incubations), at least 4 crowded plates of mixed developmental stages were used per sample. 

2. Worms were centrifuged at 2000 x g for 2 min to bring worms to the bottom of the conical and M9 buffer was removed.

NOTE: For centrifugation speed, minimum possible speed needed to pellet worms was used. Density of liquid changes throughout protocol, so speed was optimized accordingly. 

3. Worms were washed 3 times with 1 mL of M9 buffer each, then centrifuged at 2000 x g for 2 min between washes. 

4. Worms were aliquoted into 1.5 mL tubes. Total volume (M9 + worms) was no more than 100-200 µL in each tube.5. 1 mL of fresh paraformaldehyde (PFA) was added to each tube, on ice. Samples were immediately frozen at -80˚C overnight (>12h) before use.

NOTE: Worms can stay in -80˚C for long-term storage. Up to two weeks was attempted with minimal effect on quality of results.

6. Worms were fixed by thawing at room temperature for 45 minutes. This was done on a nutator for best results.

7. Worms were washed 2 times with 1 mL of PBST each. Worms were then centrifuged at 2000 x g for 2 min in between washes. 

8. Worms were incubated in 1 mL of proteinase K (100 µg/mL) for 15 minutes at 37˚C on a nutator. Proteinase K concentration and treatment time was re-optimized for each batch of proteinase K and for samples at different developmental stages. Longer incubation period will allow for better probing of adult animals, for example.

9. Worms were washed 2 times with 1 mL of PBST each. 

10. Samples were incubated in 1 mL of 2 mg/mL of glycine solution for 15 minutes on ice. 

11. Larvae were washed 2 times with 1 mL of PBST each. 

Detection :

1. Worms were incubated in 1 mL of 50% PBST / 50% probe hybridization buffer for 5 minutes at room temperature on a nutator.

NOTE: Probe hybridization buffer was warmed to room temperature before using.

2. Samples were centrifuged at 2000 x g for 2 minutes to remove the solution. 

3. Worms were pre-hybridized in 300 µL of probe hybridization buffer at 37˚C for 1 hour on a nutator.4. Probe solution was prepped by adding an optimized concentration of 1µM for each probe set stock to 200 µl of probe hybridization buffer. For robust probe sets, like *Ppa-unc-25* and *Ppa-unc-17* , 2 pmol (2 µl) was sufficient to visualize gene expression pattern. For others ( *Ppa-unc-30* and *Ppa-eat-4* ), a higher concentration of 4 pmol (4 µl) was used.5. The probe solution was then added to each sample for a total hybridization volume of 500 µL in each tube.6. Worms were incubated overnight (>12h) at 37˚C on a nutator.7. The next day, worms were washed 4 times for 15 minutes each with 1 mL of probe wash buffer at 37˚C on a nutator. Probe wash buffer was pre-heated to 37˚C.NOTE: Worms were centrifuged at 1000 x g for 2 minutes for each wash. This is a lower speed than before – gentleness was required with the samples from this step onwards.8. Worms were washed 2 times for 5 minutes each with 1 mL of 5x SSCT at room temperature on a nutator. Amplification :

1. Worms were pre-amplified with 300 µL of amplification buffer for 30 minutes at room temperature on a nutator.

NOTE: Amplification buffer was warmed to room temperature before using.2. Hairpins were cooled as follows: 10 µL of 3 µM stock was incubated at 95C for 90 seconds and then cooled to room temperature in the dark for 30 minutes (= "snap-cooling").NOTE: Hairpins h1 and h2 were provided in hairpin storage buffer from Molecular Instruments ready for snap cooling. h1 and h2 were snap cooled in separate tubes. When using multiple hairpins in double-labeling experiments, h1 hairpins were combined with other h1 hairpins, and h2 hairpins with other h2 hairpins at this step.3. Snap-cooled h1 hairpins and snap-cooled h2 hairpins were added to 200 µL of amplification buffer at room temperature to make final hairpin solution.4. The hairpin solution was added to each sample for a total amplification volume of 500 µL.5. Worms were incubated overnight (>12h) in the dark at room temperature on a nutator, either covered with foil or in a dark drawer.6. The next day, samples were washed with 1 mL of 5x SSCT at room temperature, on a nutator: first, 2 times for 5 minutes each, then 2 times for 30 minutes each, then 1 time for 5 minutes.7. Worms were resuspended in 100 µL of ProLong Gold Antifade Reagent. Samples were stored at 4˚C protected from light before microscopy.

NOTE: Samples were imaged using a standard upright compound light microscope (equipped with the necessary filters) as soon as possible for best results.

## Reagents

*Probe Hybridization Buffer, Probe Wash Buffer, and Amplification Buffer were ordered from molecularinstruments.com.*

M9 buffer

22 mM KH _2_ PO _4_

42 mM Na _2_ HPO _4_

20.5 mM NaCl

1 mM MgSO _4_

*For 1 L of solution:*

3 g of KH _2_ PO _4_

6 g of Na _2_ HPO _4_

5 g of NaCl

1 mL of 1 M MgSO _4_

Fill up to I L with ultrapure H _2_ O

Sterilize by autoclaving

4% Paraformaldehyde (PFA)

*For 40 mL of solution:*

5 mL of 32% PFA solution

4 mL of 10x PBS

Fill up to 40 mL with NF-free H _2_ O

PBST (1% Tween 20)

*For 50 mL of solution:*

5 mL of 10x PBS

500 µL of Tween 20

Fill up to 50 mL with NF-free H _2_ O

Proteinase K solution (100µg/mL)

*For 1 mL of solution:*

5 µL of 20 mg/mL proteinase K

Fill up to 1 mL with PBST

Store at -20˚C

Glycine solution (2mg/mL)

*For 50 mL of solution:*

100 mg of glycine Fill up to 50 mL with PBST

Store at 4˚C

5x SSCT (1% Tween 20) *For 40 mL of solution:*

10 mL of 20x SSC

400 µL of 10% Tween 20

Fill up to 40 mL with NF-free H _2_ O
